# Epidemiology and Pattern of Orthopedic Trauma in Children and Adolescents: Implications for Injury Prevention

**DOI:** 10.7759/cureus.39482

**Published:** 2023-05-25

**Authors:** Mahdi Mofarah Alqarni, Abdullah A Alaskari, Ahmed S AL Zomia, Abdulrahman M Moqbil, Yazeed S Alshahrani, Lama Lahiq, Shatha S Alshahrani, Ali A Alqahtani, Abdulrhman M Alqarni

**Affiliations:** 1 Department of Pediatric Orthopedics, Abha Maternity and Children Hospital, Abha, SAU; 2 College of Medicine, King Khalid University, Abha, SAU; 3 College of Medicine, Umm Al-Qura University, Al-Qunfudhah, SAU

**Keywords:** saudi arabia, management, causes, pattern, prevalence, trauma, childhood injury

## Abstract

Background: Orthopedic injuries are prevalent in children and can result in hospitalization and damage. The number of accidental injuries among children increases every year, leading to a huge burden on communities and health institutions.

Aim: This study aimed to assess the epidemiological pattern of orthopedic trauma among children and adolescents in Abha, Saudi Arabia.

Methods: A retrospective record-based study was carried out to investigate the epidemiological pattern of orthopedic trauma among children and adolescents treated at Abha Maternity and Children Hospital in Saudi Arabia, a traumatic center for pediatric patients. The study covered all children and adolescents treated at the hospital for orthopedic trauma. The parents of the children and adolescents were called to get their consent to participate in the study. The following data were extracted from the medical files: personal information, medical history, trauma-related details, management, hospitalization, and complications.

Results: A total of 295 children and adolescents were included. The mean ± standard deviation age was 6.8 ± 3.1 years old (range 1 month to 13 years). Of the patients, 186 (63.1%) were male. The most reported causes of traumas were fall from height (48.1%) and injury while playing (19.7%). The most affected body parts included the forearm (22.4%), head (21.7%), thigh (20%), and leg (10.8%). The vast majority of the children and adolescents (87.1%) had no complications.

Conclusion: The current study revealed that pediatric orthopedic injuries are not rare, and there is a higher likelihood of injuries among young male children. Fall from height and play-associated injuries are the most frequent causes.

## Introduction

Trauma is defined as “any kind of wound or penetrating/non-penetrating harm induced purposefully or inadvertently by external forces” and can occur due to road accidents, poisoning, falling, and drowning, among other causes [1,2]. Each year, trauma is responsible for more than 15,000 deaths and 50% of all reported deaths are children [2,3]. With the spread of urbanization, increasing vehicle ownership, and the upsurge in outdoor activities among children, it is likely that the incidence of trauma will continue to increase [4].

Accidents, falls (including falling from heights), sports-related injuries, assaults, burns, and drownings are the most reported causes of traumatic mortality among children [5]. Children who have permanent injuries due to these events experience long-term disability and great psychological, financial, and social burdens [6,7]. There are many factors behind higher risk of accidents among children, including their diminished risk assessment, slow reaction times, and failure to identify danger in time [8]. The epidemiology and pattern of trauma-related accidents in the pediatric population vary from country to country based on factors including the socioeconomic status, geographic location, and population-related characteristics of the specific region [9]. Childhood injuries occur most frequently at home, where young children spend most of their time. A home environment that is suitable for parents may not be appropriate for a growing child. Childhood injury prevention involves making a home as child safe as possible [10-12].

The aim of this study was to determine the prevalence of trauma in children and adolescents treated at a hospital in Abha, Saudi Arabia, the sequelae and management of this trauma, and preventative approaches to reduce trauma in this population. 

## Materials and methods

A retrospective record-based study was carried out to analyze the epidemiological pattern of orthopedic trauma among children and adolescents treated at Saudi Arabia's Abha Maternity and Children Hospital. The Ministry of Health's Asser Regional Committee for Research Ethics approved the project. The approval number is REC-12-11-2022.

The medical record system was used to identify all children and adolescents with orthopedic trauma who were treated at the Abha Maternity and Children Hospital during the study period. Patients aged 0 to 18 years old with a diagnosis of orthopedic trauma who were treated at the study hospital between 1/1/2021 and 1/1/2023 were eligible. Patients with incomplete medical records, missing data, or whose parents refused to give their child's data were excluded.

The parents of eligible patients were called to obtain their permission to participate in the study. To minimize errors, data were obtained from individuals who consented to use a pre-structured questionnaire. Personal information, such as age, gender, and nationality; medical history, including pre-existing medical conditions and medications; the type of orthopedic trauma, such as fractures, dislocations, or soft tissue injuries; the causes of the trauma, such as falls, traffic accidents, or sports injuries; the pattern of the trauma, such as open or closed fractures; and the severity of the trauma were all included in the questionnaire.

Additional information gathered included the afflicted body part(s), the type of trauma-related management and hospitalization, and the presence of trauma-related sequelae. The data were gathered by trained medical students under the supervision of an experienced orthopedic consultant.

Data analysis

The data were collected, reviewed, and then input into IBM SPSS Statistics for Windows, Version 21 (Released 2012; IBM Corp., Armonk, New York, United States). All statistical tests were two-tailed. P ≤ 0.05 was considered significant. The study variables, including personal data, medical history, epidemiological pattern of trauma, management, and complications, are presented as frequencies and percentages. The epidemiological pattern and management of trauma were compared between male and female children and adolescents with Pearson’s chi-square test for significance or the exact probability test (for small frequency distributions).

## Results

We included 295 children and adolescents. The mean ± standard deviation (SD) age was 6.8 ± 3.1 years (range 1 month to 13 years). Of these patients, 186 (63.1%) were male. Regarding chronic health problems, 5 (1.7%) children and adolescents were obese, and 1 (0.3%) each had neurological disease, diabetes mellitus, bone metabolic disease, and osteogenesis imperfecta. The remaining 290 children (96.9%) had no chronic disease (Table 1).

**Table 1:** Demographic characteristics of the children and adolescents with orthopedic trauma treated at Abha Maternity and Children Hospital, Saudi Arabia N: Number

Characteristic	N	%
Age in years		
< 3	35	11.9
3-5	66	22.4
6-9	133	45.1
10-13	61	20.7
Gender		
Male	186	63.1
Female	109	36.9
Does the child or adolescent have any diseases?		
None	286	96.9
Obesity	5	1.7
Neurological diseases	1	0.3
Bone metabolic disease	1	0.3
Diabetes mellitus	1	0.3
Osteogenesis imperfecta	1	0.3

Table 2 shows the epidemiological pattern of orthopedic trauma among the children and adolescents. We found that 91.2% of the children and adolescents had closed fractures and 92.5% had a blunt injury. The most reported causes of trauma were fall from height (48.1%), injury while playing (19.7%), car accidents (13.2%), and heavy object injuries (9.5%). Trauma was moderate among 45.8% of the children and adolescents, serious but not life-threatening among 40.3%, and life-threatening among 2.4%. For the vast majority of the children and adolescents, the trauma affected only one part of their body (94.6%). The most affected body parts included the forearm (22.4%), head (21.7%), thigh (20%), and leg (10.8%). There was a uniform incidence of trauma for each season of the year. There was no significant difference in the trauma pattern between male and female children and adolescents. 

**Table 2:** Epidemiological pattern of orthopedic trauma among the children and adolescents treated at Abha Maternity and Children Hospital, Saudi Arabia No: Number The P-value was determined by using the Pearson’s χ2 test, except when marked with * (exact probability test)

Trauma	Total		Gender				P
			Male		Female		
	No	%	No	%	No	%	
What is the trauma?							0.222^*^
Dislocation	19	6.4	9	4.8	10	9.2	
Closed fracture	269	91.2	171	91.9	98	89.9	
Open fracture	3	1.0	2	1.1	1	0.9	
Others	4	1.4	4	2.2	0	0.0	
Type of trauma							0.953
Blunt injury	273	92.5	172	92.5	101	92.7	
Penetrating injury	22	7.5	14	7.5	8	7.3	
Cause of trauma							0.276^*^
Car accident	39	13.2	23	12.4	16	14.7	
Bicycle injury	8	2.7	7	3.8	1	0.9	
Fall from height	142	48.1	89	47.8	53	48.6	
Fight	14	4.7	11	5.9	3	2.8	
Injury while playing	58	19.7	40	21.5	18	16.5	
Heavy object	28	9.5	13	7.0	15	13.8	
Run over	2	0.7	1	0.5	1	0.9	
Head trauma during seizure	1	0.3	1	0.5	0	0.0	
Pathological	3	1.0	1	0.5	2	1.8	
Severity of the trauma							0.159
Minor	34	11.5	24	12.9	10	9.2	
Moderate	135	45.8	89	47.8	46	42.2	
Serious, not life threatening	119	40.3	67	36.0	52	47.7	
Serious, life threatening	7	2.4	6	3.2	1	0.9	
How many parts of the body were affected by the trauma?							0.438^*^
1	279	94.6	174	93.5	105	96.3	
2	14	4.7	10	5.4	4	3.7	
≥ 3	2	0.7	2	1.1	0	0.0	
Which part(s) of the body were affected by the trauma?							0.237^*^
Head	64	21.7	45	24.2	19	17.4	
Face	15	5.1	11	5.9	4	3.7	
Mandible	2	0.7	2	1.1	0	0.0	
Neck	1	0.3	1	0.5	0	0.0	
Arm	14	4.7	9	4.8	5	4.6	
Forearm	66	22.4	41	22.0	25	22.9	
Hand	30	10.2	14	7.5	16	14.7	
Elbow	2	0.7	2	1.1	0	0.0	
Wrist	1	0.3	1	0.5	0	0.0	
Thigh	59	20.0	35	18.8	24	22.0	
Leg	32	10.8	17	9.1	15	13.8	
Ankle	2	0.7	1	0.5	1	0.9	
Foot	7	2.4	7	3.8	0	0.0	
Which season of the year did the trauma occur?							0.436
Spring	72	24.4	42	22.6	30	27.5	
Summer	67	22.7	41	22.0	26	23.9	
Autumn	78	26.4	55	29.6	23	21.1	
Winter	78	26.4	48	25.8	30	27.5	

Table 3 shows the management and hospitalization of orthopedic trauma among the children and adolescents. Overall, 87.1% of the children and adolescents were taken to the hospital by another person (i.e., not in an ambulance). In terms of timing, 50.5% of the children and adolescents went to the hospital within 1-2 hours after trauma and 32.5% went to the hospital within less than one hour. We found that 68.1% of the patients needed to be hospitalized after the trauma; the length was for 2-3 days for 45.8% of the children and adolescents and one day for 32.3%. Closed reduction with casting was performed for 35.9% of the children and adolescents, 16.6% underwent closed reduction (traction), and 12.5% underwent open reduction and internal fixation. In addition, 27.8% of the children and adolescents had no intervention. They only underwent observation. Follow-up was done for 70.2% of the children and adolescents; 82.7% of these children and adolescents received medications (antibiotics (55.3%) and analgesics (54.6%)). There was a significant difference in hospitalization after trauma between males and females (72.6% for males and 60.6% for females, P = 0.032).

**Table 3:** Management and hospitalization of orthopedic trauma among the children and adolescents treated at Abha Maternity and Children Hospital, Saudi Arabia No: Number The P-value was determined by using the Pearson’s χ2 test, except when marked with $ (exact probability test). An asterisk indicates a significant difference between males and females

Management	Total		Gender				P
			Male		Female		
	No	%	No	%	No	%	
How was the patient taken to the hospital?							0.730
Ambulance	38	12.9	23	12.4	15	13.8	
Someone else	257	87.1	163	87.6	94	86.2	
Time between trauma and arrival to the hospital (hours)							0.787
< 1	96	32.5	64	34.4	32	29.4	
1-2	149	50.5	92	49.5	57	52.3	
3-6	41	13.9	24	12.9	17	15.6	
> 6	9	3.1	6	3.2	3	2.8	
Did the patient need to be hospitalized after the trauma?							0.032*
Yes	201	68.1	135	72.6	66	60.6	
No	94	31.9	51	27.4	43	39.4	
If yes, hospitalized for how many days?							0.720
1	65	32.3	45	33.3	20	30.3	
2-3	92	45.8	59	43.7	33	50.0	
4-6	30	14.9	20	14.8	10	15.2	
≥ 7	14	7.0	11	8.1	3	4.5	
What procedure did the patient receive?							0.184^$^
Closed reduction (casting)	106	35.9	65	34.9	41	37.6	
Closed reduction (traction)	49	16.6	24	12.9	25	22.9	
Open reduction and internal fixation	37	12.5	25	13.4	12	11.0	
Depressed fracture elevation	4	1.4	4	2.2	0	0.0	
EPH[PRS1] evacuation	3	1.0	2	1.1	1	0.9	
Left against medical advice	3	1.0	1	0.5	2	1.8	
Soft neck collar	1	0.3	1	0.5	0	0.0	
Cranioplasty	1	0.3	0	0.0	1	0.9	
Nasal bone	9	3.1	6	3.2	3	2.8	
Only observation	82	27.8	58	31.2	24	22.0	
Regular follow-up after trauma?							0.898
Yes	207	70.2	131	70.4	76	69.7	
No	88	29.8	55	29.6	33	30.3	
Received medications?							0.435^$^
None	51	17.3	30	16.1	21	19.3	
Analgesic	161	54.6	106	57.0	55	50.5	
Antibiotic	163	55.3	100	53.8	63	57.8	
Anticonvulsant	14	4.7	7	3.8	7	6.4	
Antiemetic	6	2.0	5	2.7	1	0.9	
Omeprazole	9	3.1	8	4.3	1	0.9	
Decongestant	5	1.7	3	1.6	2	1.8	

Table 4 shows the complications of orthopedic trauma among the children and adolescents. The most reported complications were inability to move the affected area (4.1%), convulsions (3.7%), and LOC[PRS1] (3.1%). The vast majority of children and adolescents (87.1%) had no complications.

**Table 4:** Complications of orthopedic trauma among children and adolescents treated at Abha Maternity and Children Hospital, Saudi Arabia No: Number; EDH: extradural hematoma; LOC: loss of consciousness The P-value was determined by using the exact probability test

Complications	Total		Gender				P
			Male		Female		
	No	%	No	%	No	%	
Bleeding	1	0.3	1	0.5	0	0.0	0.183
Inability to move the affected area	12	4.1	5	2.7	7	6.4	
Convulsions	11	3.7	8	4.3	3	2.8	
EDH	4	1.4	4	2.2	0	0.0	
LOC	9	3.1	4	2.2	5	4.6	
Leptomeningeal cyst	1	0.3	0	0.0	1	.9	
None	257	87.1	164	88.2	93	85.3	

## Discussion

Understanding the pattern of injuries among children is important in reducing morbidity and mortality through targeted prevention efforts. According to a study published in the Journal of Pediatric Surgery, pediatric injury patterns vary by age and can be identified by using large national databases [13]. According to the United States Centers for Disease Control and Prevention (CDC), injury is still the leading cause of death for children and teens in the United States, with more than 7,000 children and teens aged 0-19 years dying because of unintentional injuries in 2019 [14]. Another report from the CDC on the patterns of unintentional injuries among children in the US from 2000 to 2006 defined eight subgroups based on the mechanism of injury: motor-vehicle crash occupants, pedestrian and cycle injuries, falls, child abuse, gunshot and stab wounds, burns, poisonings, and foreign body ingestions or aspirations [15].

In the present study, we found that most of the injured children were < 10 years old and were male. Regarding the injury pattern, most children had blunt orthopedic injuries with closed fractures mainly due to a fall from height (due to their young age) and injury during playing (as most of them were boys who may be outdoors for a long time). Sharma et al. reported that falls were the most common cause of pediatric injuries, followed by road traffic accidents [16]. Similarly, Hyder et al. stated that more than one-third of all injuries were due to falls in children < 5 years old [17]. A study in Nepal revealed that falls were the most common injury, reported among two-thirds of the study participants [18]. Most injuries were moderate and not life-threatening.

In the current study, the upper extremities, head, and lower extremities were the most frequent sites for orthopedic injuries. Lien reported similar findings [19]. In India, Singh et al. found that more than half of the children who experienced orthopedic injuries were < 5 years old and mainly male [20]. Moreover, the right extremities were more commonly reported as the site of injury. Upper limb injuries were most common followed by lower limb and pelvic injuries. Very few patients had isolated spinal injuries. More than half of patients had a history of falling, followed by road-traffic-accident-related injuries. Likewise, Kulshrestha et al. [21] and Verma et al. [22] reported that boys experienced higher rates of musculoskeletal injuries compared with girls. Rasmussen et al. indicated a high incidence of orthopedic trauma among children during the COVID-19 pandemic due to playing in unsuitable home environments [23]. In Iran, Ghaffari et al. found that the vast majority of the fractures were in the upper limbs, with less than one-fifth observed in the lower limbs [24]. Moreover, the most common site of trauma was at home, and the most frequent mechanism in boys and girls was falling (42.3%). Finally, the most common season for injuries was the fall (44.6%). Most orthopedic trauma (56.7%) occurred in children and adolescents with a normal body mass index: Only 8.29% of fracture cases were related to obese or overweight patients aged 2-16 years. Nakaniida et al. reported that the most common injuries were femur fractures in young children and vertebral fractures in adolescents [25]. The most common injuries requiring hospitalization were femur and humerus fractures. In Saudi Arabia, Albedewi et al. found that most pediatric injuries occurred among boys, and the leading cause of fractures was falls (37.9%), followed by MVC[PRS1] (21.5%) [26]. In addition, the weighted mortality rate was 5.2% for overall injuries and 8.3% for fractures. Abed et al. found that the vast majority of the injured children were boys, but interestingly more girls experienced fracture injuries than boys [27]. The most common causes of injury included falling (38.5%), followed by road traffic accidents (26.1%). Moreover, the head and neck areas were the most affected sites, contributing to 39.1% of all injuries.

Regarding management and outcomes, we found that most of the children were taken to the hospital by another person (i.e., not by an ambulance), and half of them went to the hospital within 1-2 hours after trauma. About two-thirds of the patients needed to be hospitalized after the trauma. Closed reduction with casting and that with traction were the most applied treatment approaches, and some patients underwent open reduction and internal fixation. Considering the clinical outcome (complications), the most reported complications were the inability to move the affected area (4.1%), convulsions (3.7%), and LOC (3.1%). Of note, most of the patients had no complications. Adesunkanmi et al. reported that road traffic injuries and burns accounted for the greatest number of complications [28].

Limitations

Because this study was conducted in a particular location, the findings may not be generalizable to other contexts or groups. This center's patient demographics and resource availability may not be representative of other centers, limiting the findings' application to larger groups. Furthermore, the study may have missed certain important aspects that could affect patient outcomes in other contexts, such as differences in treatment protocols, patient comorbidities, or environmental conditions. As a result, caution should be exercised when projecting the findings to other settings or populations, and additional studies may be required to establish the results' generalizability.

Recommendations

Based on the study's findings, it is suggested that more efforts be made to reduce the frequency of trauma among youngsters. This could involve raising parents' and caregivers' awareness of the possible hazards of falls and other injuries, as well as giving education and tools to assist prevent these accidents. Furthermore, future research should concentrate on identifying specific risk factors for pediatric orthopedic trauma and creating tailored therapies to mitigate these risks. Overall, these measures may contribute to a reduction in the burden of pediatric orthopedic injuries on communities and healthcare institutions.

## Conclusions

In conclusion, we have revealed that childhood orthopedic injuries in Abha, Saudi Arabia are not rare and have a higher likelihood to occur among younger male children. Fall from height and play-associated injuries were the most frequent causes, and most injuries were blunt, including closed fractures. The upper extremities, head, and lower extremities were the most common trauma sites, and the injuries were usually not severe. The hospitalization rate after injury was high but most cases needed non-surgical intervention. Moreover, there were very few complications among the children and adolescents. Most of the reported injuries could have been prevented by improving the safety of the environment. In addition, enhancing awareness through public health campaigns about the value of supervision by guardians may significantly minimize the chances of accidental injuries.
